# Supplementary material for: Cuticular modified air sacs underlie white coloration in the olive fruit fly, Bactrocera oleae
Source: Commun Biol. 2021 Jul 16;4:881. doi: 10.1038/s42003-021-02396-4 (PMC8285419; doi:10.1038/s42003-021-02396-4)
Supplement: Supplementary file 2 — Supplementary Information [file 42003_2021_2396_MOESM2_ESM.pdf]

Cuticular modified air sacs underlie white coloration in the olive fruit fly, *Bactrocera oleae*

Manuela Rebora, Gianandrea Salerno, Silvana Piersanti, Alexander Kovalev and Stanislav Gorb

Supplementary Table 1 Parameters of statistical analysis of reflectance spectra from scutellum and dark areas on thorax of *Bactrocera oleae* (n = 5 males; n = 5 females) (see Fig. 10).

Factors	d.f.	*F*	*P*
3-way ANOVA for reflectance spectra in the range 550-700 nm			
Sex	1	3.30	0.076
Thorax color	1	384.12	<0.001
Angle	2	5.99	0.005
Sex x Thorax color	1	8.19	0.006
Sex x Angle	2	0.34	0.713
Thorax color x Angle	2	12.90	<0.001
Sex x Thorax color x Angle	2	0.57	0.568
Error	48		
3-way ANOVA for reflectance spectra in the UV range			
Sex	1	0.001	0.973
Thorax color	1	48.66	<0.001
Angle	2	4.02	0.024
Sex x Thorax color	1	0.56	0.460
Sex x Angle	2	0.92	0.406
Thorax color x Angle	2	2.18	0.124
Sex x Thorax color x Angle	2	2.10	0.133
Error	48		

Supplementary Table 2 Ultraviolet (330-400 nm) reflection (mean ± standard error). The difference between mean values marked with the same sign is not statistically significant (see Table 1).

Location \ Angle	0°	30°	45°
white spot	0.038^{*}±0.003	0.042^{*}±0.004	0.036^{*}±0.005
dark thorax	0.054^{x}±0.006	0.081^{+}±0.008	0.072^{x+}±0.007

Supplementary Table 3 Reflection of the visible light (550-700 nm) from the white spot (mean ± standard error). The difference between mean values marked with the same sign is not statistically significant (see Table 1). The mean reflection from the dark area on thorax was 0.078±0.005.

Sex \ Angle	0°	30°	45°
♂	0.246^{*}±0.008	0.231^{*}±0.009	0.185±0.010
♀	0.218^{+}±0.013	0.197^{+}±0.012	0.155±0.018

Supplementary Figure 1. Longitudinal sections of the scutellum of the pharate adult of *Bactrocera oleae* visualised with TEM.

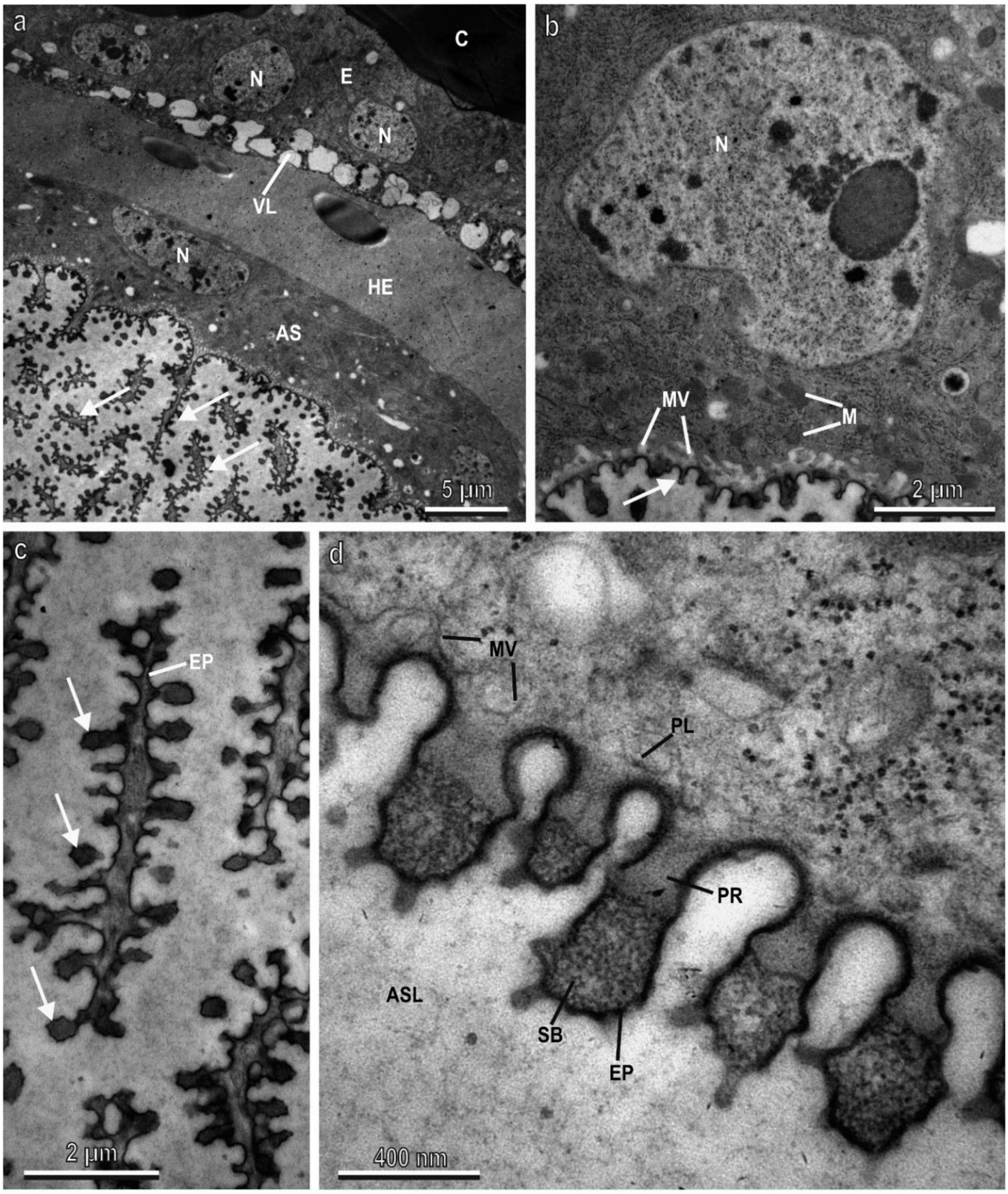

a, General view showing the transparent cuticle (C), the epidermis (E), the vesicular layer (VL), the haemolymph (HE) and the air sac (AS) constituted of a monolayer of big cells with arborisations (arrows). N, nuclei; **b**, Detail of (a) showing one of the cells of the air sac with a big nucleus (N), and numerous mitochondria (M). Note the apical border with plaques at the tip of microvilli (MV) secreting the cuticular layer (arrow) lining the lumen of the air sac; **c**, Detail of **(a)** showing the developing arborisations constituted of cytoplasm showing a thin electron-dense epicuticular layer (EP) and spiny beds (arrows); **d**, Detail of **(b)** showing the apical border of the air sac cells with plaques (PL) at the tip of microvilli (MV) secreting the epicuticular layer (EP) and the procuticle (PR) of the developing spiny beads (SB) rich of granules. Note that the beads are located at the apex of cuticular stems. ASL, air sac lumen.

Supplementary Figure 2. Frontal sections of the scutellum of the just emerged adult of *Bactrocera oleae* visualised with TEM.

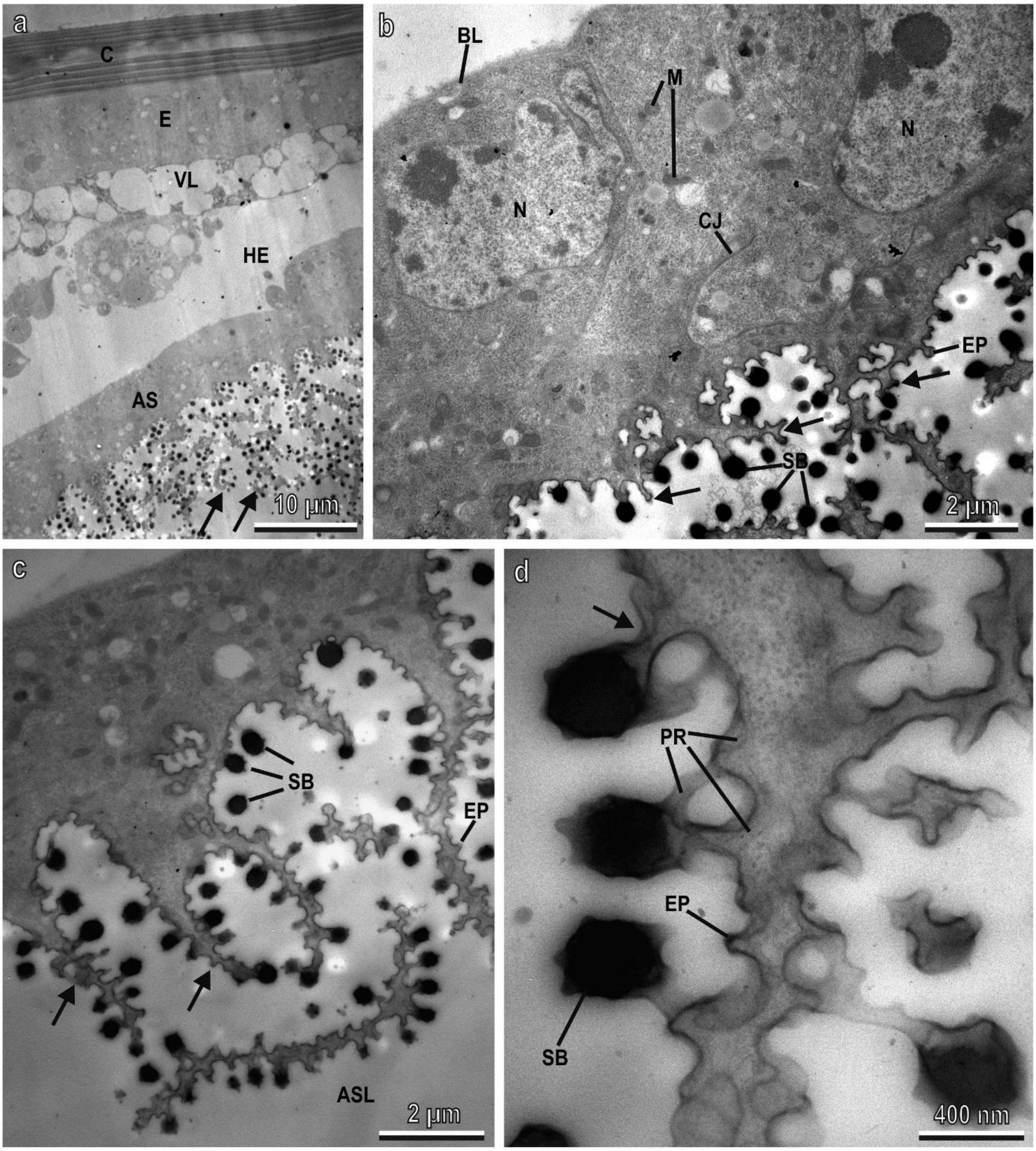

a, General view showing the multi-layered transparent cuticle (C), the epidermis (E), the vesicular layer (VL), the haemolymph (HE) and the air sac (AS) constituted of a monolayer of big cells with arborisations (arrows); **b**, Detail of **(a)** showing the monolayer of big cells of the air sac. BL; basal lamina, CJ, cell junctions, EP, epicuticular layer, N, nuclei, M, mitochondria. Arrows point out the arborisations with spiny beads (SB); **c**, Arborisations (arrows) constituted of cytoplasm showing a thin epicuticular layer (EP) and very electron-dense spiny beads (SB); **d**, Detail of **(c)** showing the fully developed spiny beads (SB) appearing very electron-dense. Note that the cuticular stems of the beads (arrow) appear more thin and elongated in comparison with those of the pharate adult. EP, epicuticular layer; PR, procuticle.

Supplementary Figure 3. Longitudinal sections of the scutellum of the adult *Bactrocera oleae* visualised with TEM.

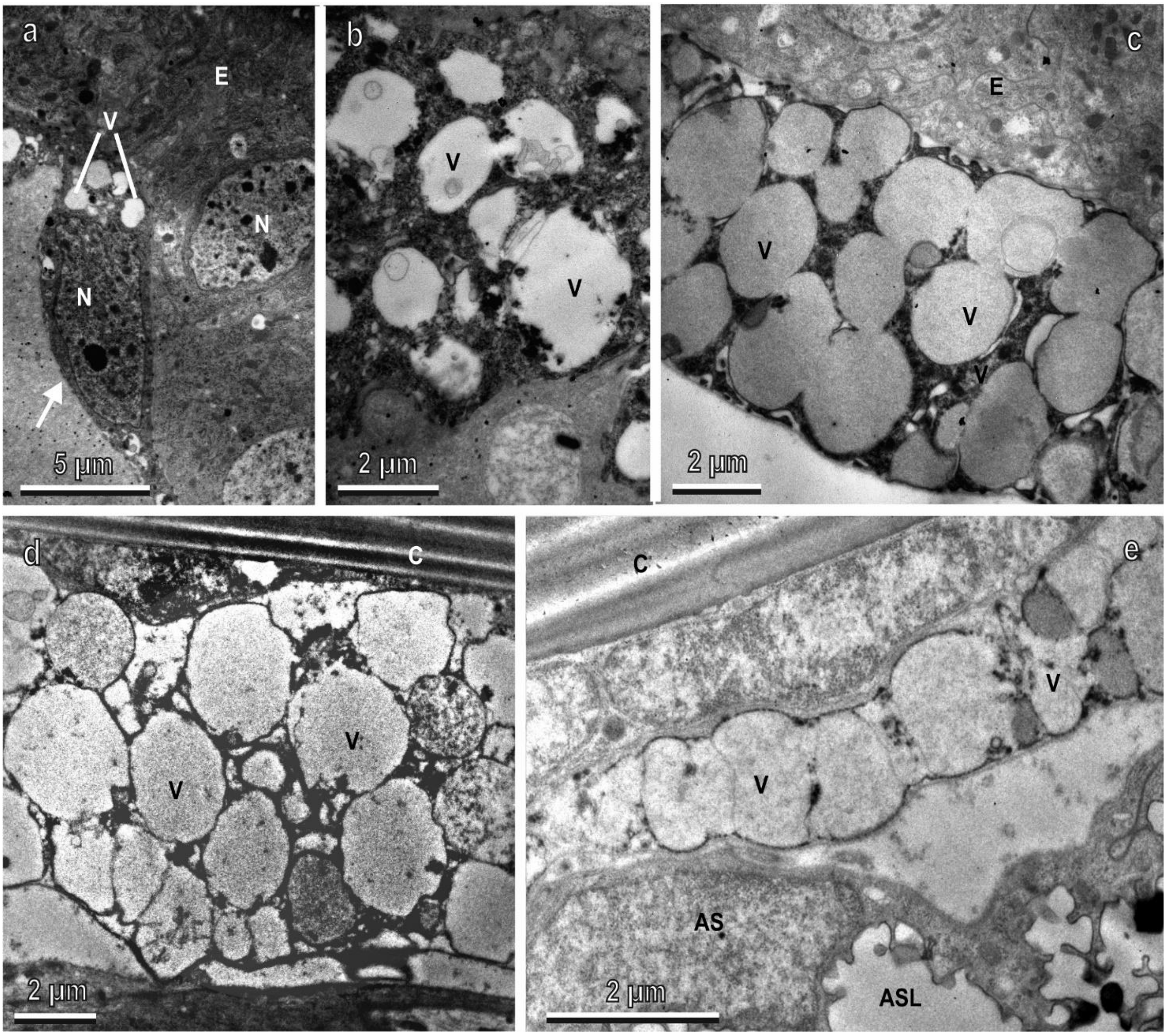

Note the development of the vesicular layer in the pharate adult **(a,b)**, in the just emerged adult **(c)** and in the ten days old female **(d)** and male **(e)**. AS, air sac; C, cuticle; E, epidermis; V, vesicles.

Supplementary Figure 4. Dorsal view of the scutellum of the adult of *Bactrocera oleae* in fluorescence light microscope with an excitation filter 365 nm, chromatic beam splitter FT 395 nm, emission 397 nm.

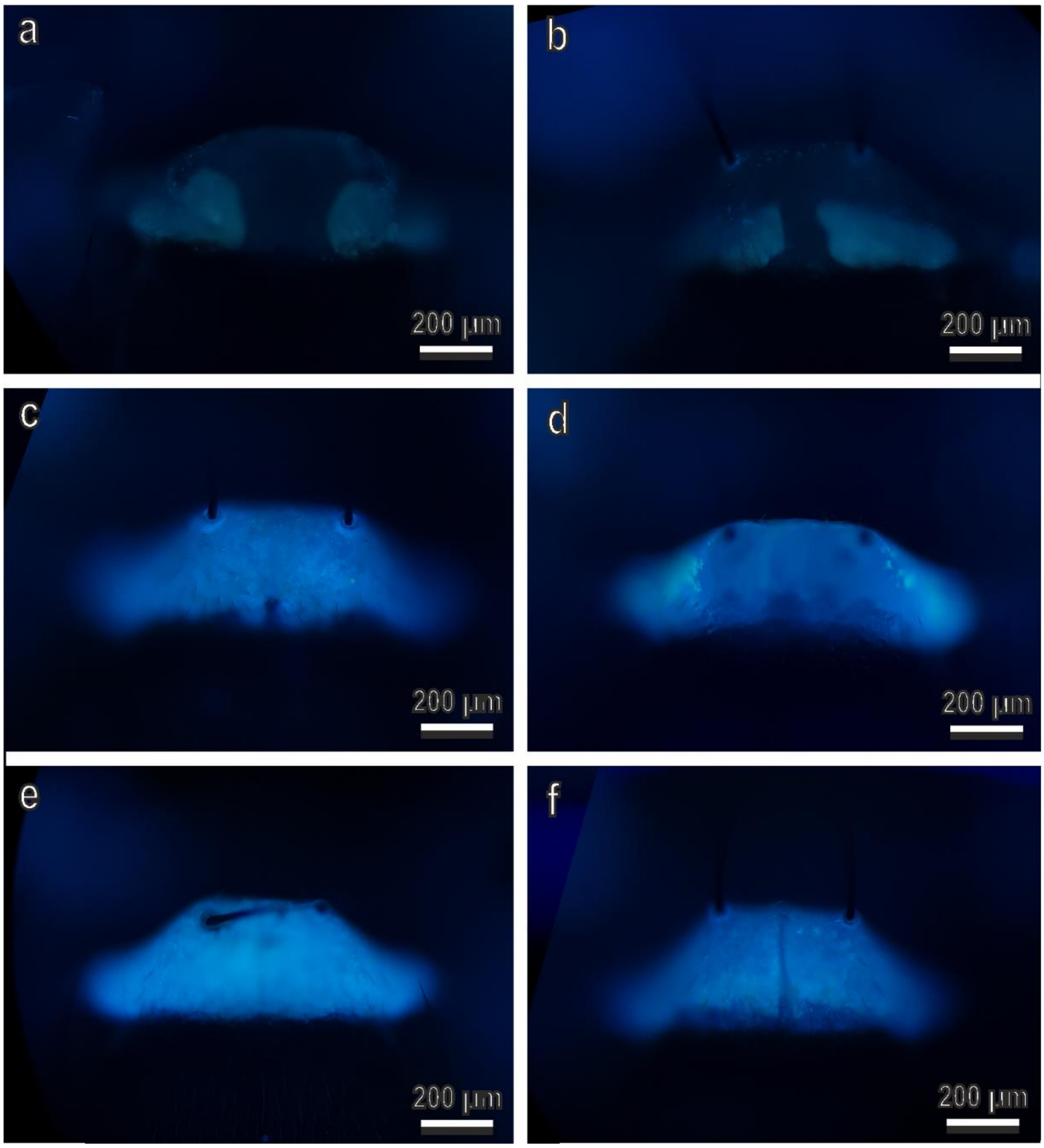

Note the UV-induced fluorescence of the scutellum in the three days old female (C), in the three days old male **(d)**, in the ten days old female **(e)**, in the ten days old male **(f)**. The UV-induced fluorescence is not present in the just emerged female (a) and male (b).

Supplementary Figure 5. Dorsal view of the scutellum of the adult (ten days old) of *Bactrocera oleae* in fluorescence light microscope.

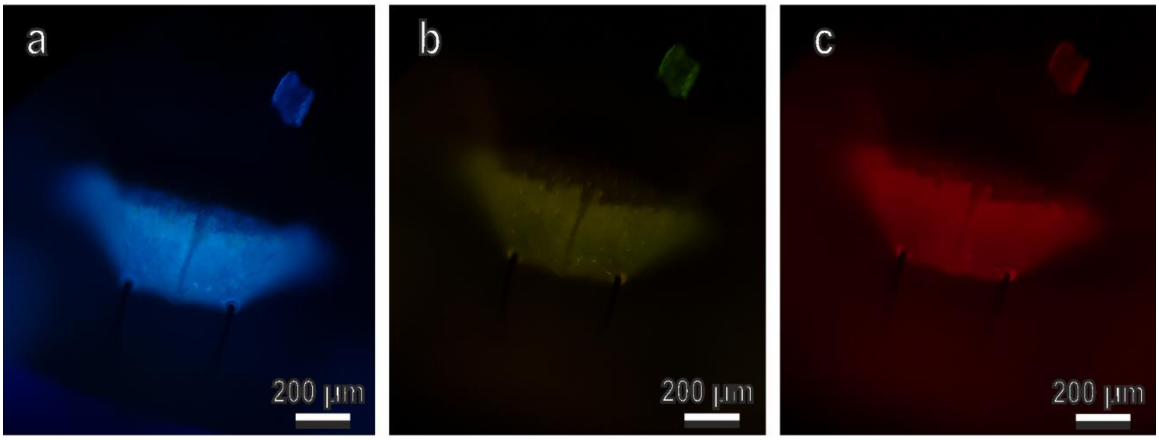

Fluorescence with an excitation filter 365 nm, chromatic beam splitter FT 395 nm, emission 397 nm (**a**), with an excitation filter 450–490 nm, chromatic beam splitter FT 510 nm, emission 520 nm (**b**), and with an excitation filter 546 nm, chromatic beam splitter FT 580 nm, emission 590 nm (**c**). Note in (a) the strong UV-induced fluorescence of the scutellum.

Supplementary Figure 6. Dorsal view of the dissected thorax showing the air sacs located inside the thorax (usually covered by dark cuticle) and intact scutellum (with transparent cuticle) of the adult (ten days old) of *Bactrocera oleae*.

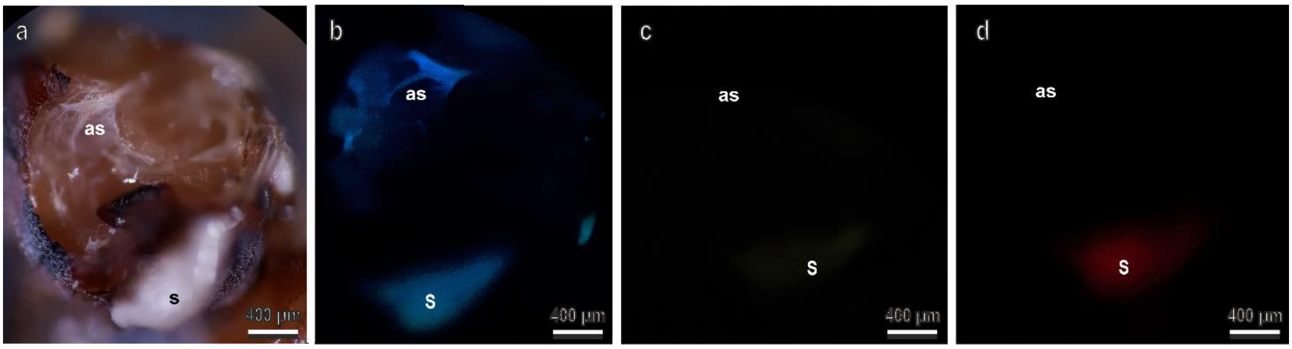

Thorax in bright-field light microscope **(a)** and in fluorescence light microscope **(b–d)**. Emitted fluorescence with an excitation filter 365 nm, chromatic beam splitter FT 395 nm, emission 397 nm (**b**), with an excitation filter 450–490 nm, chromatic beam splitter FT 510 nm, emission 520 nm (**c**), and with an excitation filter 546 nm, chromatic beam splitter FT 580 nm, emission 590 nm (**d**). Note in (a) the strong UV-induced blue autofluorescence of the air sacs (AS) located inside the thorax and of the intact scutellum (S).

Supplementary Figure 7. Reflection spectra smoothing using Savitzky-Golay filter.

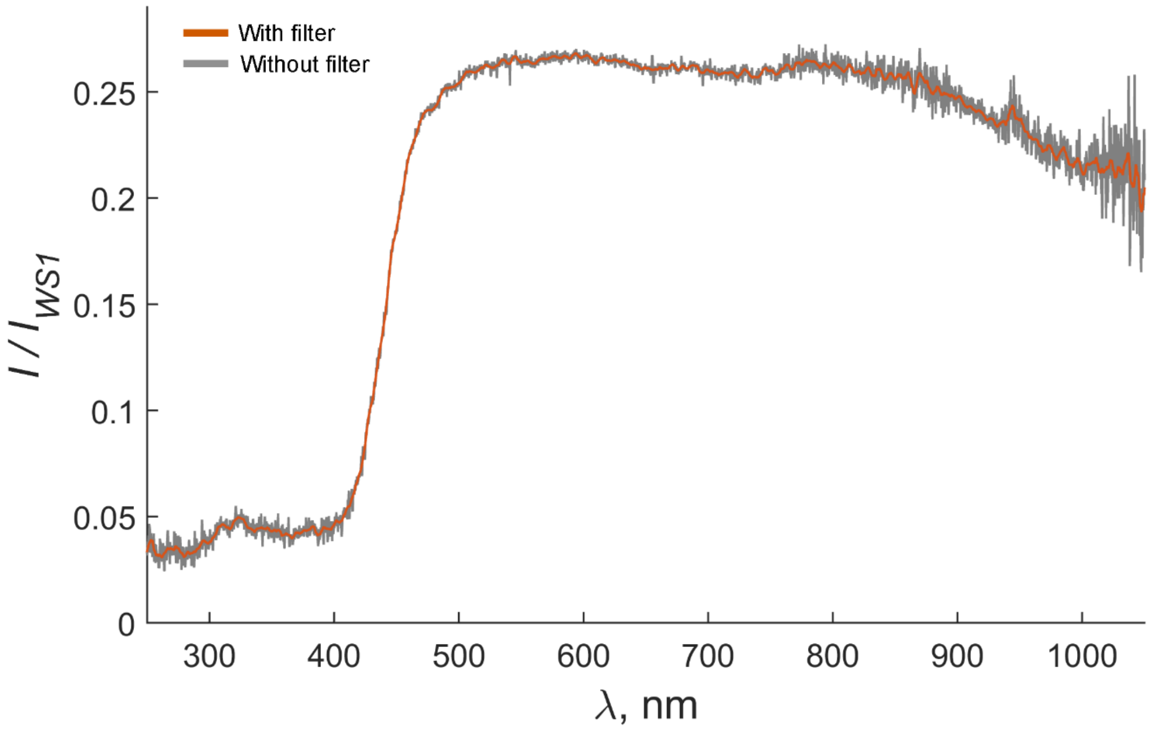

A typical normalized reflection spectrum from a thoracic white patch of *Bactrocera oleae* male is shown as a grey line. The spectrum was measured at 45° illumination and 0° detection. The spectrum was normalized on the reflection from the white standard (WS1) at 45° illumination and 45° detection. The red line represents the reflection spectrum after smoothing it using Savitzky-Golay filter with 4-th order polynomials over 12 nm windows**.**
