# Supplementary material for: Cuticular modified air sacs underlie white coloration in the olive fruit fly, Bactrocera oleae
Source: Commun Biol. 2021 Jul 16;4:881. doi: 10.1038/s42003-021-02396-4 (PMC8285419; doi:10.1038/s42003-021-02396-4)
Supplement: Supplementary file 3 — Description of Additional Supplementary Files [file 42003_2021_2396_MOESM3_ESM.pdf]

Description of Additional Supplementary Files

File Name: Supplementary Data 1
Description: Source data for Fig. 7

File Name: Supplementary Data 2
Description: Source data for Fig. 8i

File Name: Supplementary Data 3
Description: Source data for Fig. 8j

File Name: Supplementary Data 4
Description: Source data for Supplementary Fig. 7
